# A Clinical Introduction to the Practice of Auscultation

**Published:** 1854-12

**Authors:** 


Art. VIII.—*A Clinical Introduction to the Practice of Auscultation, and other modes of Physical Diagnosis, in Diseases of the Lungs and Heart.* By H. M. Hughes, M. D.; Fellow of the Royal College of Physicians; Assistant Physician to Guy's Hospital, etc. Second American, from the second and revised English edition. Philadelphia: Blanchard and Lea. 1854. pp. 304, 16 mo.

---

The diagnosis of diseases of the chest is invested with peculiar interest both from its difficulty and its importance. The means of instruction in this department are usually so deficient in the medical colleges of this country that the author who simplifies the subject and thereby brings it within the reach of the largest number of medical inquirers entitles himself to the especial thanks of the profession.

The work of Dr. Hughes accomplishes this in an eminent degree. It was written for students (whether old or young) for the purpose of preparing and helping them to examine for themselves. In style it is simple, plain and colloquial; devoid of "erudite mystery," and intelligible to all. In matter it is as full as an egg is of meat. We hail the demand for a second edition as an evidence of progress in the right direction, and heartily recommend it to the student of auscultation and percussion as an excellent and safe guide.

There are, it is true, some doctrines advanced by Dr. Hughes which in view of Skoda's opinions we do not feel altogether willing to underwrite, but taken as a whole, the work is sound and orthodox.

Our readeri will endorse the doctrine of the following extract which is all that we have room for:

"It is essential to the successful prosecution of auscultation and percussion that their value should be fully, but at the same time, fairly estimated. If the individual employing them be influenced merely by fashion, and labor under the misconception that they are in reality of little importance to the formation of a correct diagnosis, it is highly probable, that in his hands at least, they will prove so. If, on the other hand, the student conceive that by their use he will be enabled, in every case, to determine the exact condition of a diseased organ, he will be most assuredly liable to much disappointment. They should never be, and there is no valid reason why they ever should be, employed to the exclusion of the other modes of investigation. When the history, and the general or constitutional symptoms of disease, have been inquired into with the same minuteness, and interest as in other cases; then, but usually not till then, should percussion and auscultation be employed, or then, at any rate, and not till then, should any deductions be made from the indications afforded by the physical signs. To a neglect, or comparative disregard of the history, and ordinary symptoms of disease, and a too implicit confidence in their highly important art, it is probable that some of the errors of even accomplished auscultators are to be attributed. There is, however, I repeat, no valid reason why the auscultator should not use with equal diligence, and be equally skilled in the employment of all other modes of investigation, as the individuals who discard the stethoscope, and make no use of the ear in forming their diagnosis, and why he should not enjoy the advantage of his art *in addition* to all those possessed by others. Let, then, auscultation and percussion be regarded as ancillary only to other, or rather as two among many, modes of investigating disease; as two stout and strong additional strings to our bow; albeit the information they afford, is, in some cases, equal, if not superior, to that derived from all other modes combined."

